# Supplementary material for: Correlation between choroidal structure and smoking in eyes with central serous chorioretinopathy
Source: PLoS One. 2021 Mar 23;16(3):e0249073. doi: 10.1371/journal.pone.0249073 (PMC7987178; doi:10.1371/journal.pone.0249073)
Supplement: Attachment — Submitted filename: Response to Prof Vavvas2016.docx [file pone.0249073.s003.docx]

Dear Prof. Demetrios G. Vavvas

We appreciate you and the reviewers for the constructive and insightful comments. Please find our enclosed point-by-point responses to the reviewers’ comments. We hope that this revision will meet your expectations, and that our revised manuscript will better suit for publication on PLOS ONE.

Reviewer #1: 1. It has been reported that the choroidal thickness and choroidal vascular index are significantly different between classic and chronic CSC. However, the authors did not state the distribution of the CSC types in smoking and non-smoking groups. The distribution might affect the main results in this study.

Thank you for the critical and constructive suggestion. In the smoking group, 10 eyes were classic CSC and 35 eyes were chronic CSC. In non-smoking group, 9 eyes were classic and 21 eyes were chronic CSC. To analyze the relationship between smoking status and CSC type (classic or chronic), the chi-squared test was conducted. As a result, there was no significant relationship between smoking and CSC type (p=0.63), which would imply that the distribution of the CSC type has only a negligible effect on the current results. We have revised our manuscript in the Results section.

2. There are no evidences supporting the following statement in the discussion: luminal area increase in the smoking group was accompanied by an increase in the stromal area as well because of the increased choroidal vascular permeability in CSC patients. Why was the luminal area increased in the smoking group? Moreover, in the response letter, the authors mentioned that increased choroidal thickness with concomitant decrease in CVI was reportedly observed in some inflammatory diseases, which might support their hypothesis. However, the pathophysiology in such inflammatory diseases is quite different from that in CSC.

Thank you for the insightful comment. We added this speculation, because we speculated that the luminal area increase in the smoking group was accompanied by an increase in the stromal area due to the increased choroidal vascular permeability, basing on a previous report by Wei et al. in which it was suggested that the increased stromal area in smoking group may be attributed to a chronic proinflammatory response (Ref 12). Nonetheless, we now totally agree with the reviewer’s comment. Furthermore, in the current study, we did not clarify this hypothesis. Moreover, we also agree with the reviewer’s comment that the pathophysiology of CSC is different from that of inflammatory disease. Thus, the too much speculative description in the Discussion is now deleted in the revised manuscript.

Reviewer #2: Dear Editor,

Thank you for this opportunity to review the revised manuscript entitled “Correlation between choroidal structure and smoking in eyes with central serous chorioretinopathy”.
All the comments are properly addressed. While responding to a very good point from reviewer 1 authors investigated the incidence of CVH as assessed by ICGA. Would suggest including it in the manuscript ( main or supplement).

Thank you for the insightful comment. We have added the following sentences, according to the reviewer’s recommendation.

Results

“Forty-one of 45 smoking patients (91.1%) had CVH and 23 of 26 non-smoking patients (88.5%) had CVH. The prevalence of CVH was numerically higher in smoking group than that in non-smoking group, however, the difference was not statistically significant.”

Discussion

“Recently, CSC has been considered as one of the pachychoroid spectrum diseases with focal or diffuse increase in choroidal thickness, dilated large choroidal vessels, and CVH. In pachychoroid spectrum diseases including CSC and polypoidal choroidal vasculopathy, CVH are associated with an increased SFCT and an increased CVI (23-25). In the current study, the prevalence of CVH was compared between the smoking and non-smoking groups in eyes with CSC. As a result, the prevalence of CVH was higher in smoking group than in non-smoking group, however this difference was not significant. This may be because the frequency of CVH is high in eyes with CSC in general, which masked the difference between the smoking and non-smoking groups. It should be noted that the reason why a smoking habit causes the decreased CVI still remains unclear, and further studies are needed shedding light on this issue.”
